# The Notable Role of Telomere Length Maintenance in Complex Diseases

**DOI:** 10.3390/biomedicines12112611

**Published:** 2024-11-15

**Authors:** Jiahui Lv, Xinmiao Zhao, Linjie Zhao, Chengjun Gong, Wanjie Zheng, Li Guo, Jun Wang, Tingming Liang

**Affiliations:** 1 State Key Laboratory of Organic Electronics and Information Displays, Institute of Advanced Materials (IAM), Nanjing University of Posts and Telecommunications, Nanjing 210023, China; 1024233707@njupt.edu.cn (J.L.); 1222014425@njupt.edu.cn (X.Z.); 1223014120@njupt.edu.cn (L.Z.); 1223014119@njupt.edu.cn (C.G.); 1024233720@njupt.edu.cn (W.Z.); lguo@njupt.edu.cn (L.G.); 2 Jiangsu Key Laboratory for Molecular and Medical Biotechnology, School of Life Science, Nanjing Normal University, Nanjing 210023, China

**Keywords:** telomere length, telomerase, cancer, neurodegenerative diseases, reproductive health

## Abstract

Telomere length function serves as a critical biomarker for biological aging and overall health. Its maintenance is linked to cancer, neurodegenerative conditions, and reproductive health. This review mainly examines genetic variations and environmental influences on telomere dynamics, highlighting key regulatory genes and mechanisms. Advances in telomere measurement methodologies are also reviewed, underscoring the importance of precise telomere assessment for disease prevention and treatment. Telomerase activation offers potential for cellular lifespan extension and anti-aging effects, whereas its inhibition emerges as a promising therapeutic approach for cancer. Regulatory mechanisms of tumor suppressor genes on telomerase activity are analyzed, with a comprehensive overview of the current state and future potential of telomerase inhibitors. In addition, the association between telomeres and neurodegenerative diseases is discussed, detailing how telomere attrition heightens disease risk and outlining multiple pathways by which telomerase protects neurons from damage and apoptosis.

## 1. Introduction

Telomere length maintenance plays a pivotal role in human health, as telomeres—unique structures at chromosomal ends—undergo progressive shortening with each cell division and are closely linked to cellular senescence and the onset of various diseases. Stabilizing telomere length can delay cellular aging and lower disease risk, thereby supporting overall health. Adopting a healthy lifestyle, including a balanced diet, regular exercise, and the avoidance of harmful habits, aids in preserving telomere length and promoting well-being. Thus, the maintenance of telomere length is essential for disease prevention and slowing the aging process.

The intricate association between telomeres and disease progression is a significant research focus. Telomeres, consisting of DNA repeats at chromosomal termini, primarily function to safeguard chromosomes from degradation and prevent chromosomal end-to-end fusion [1]. With successive cell divisions, telomeres gradually shorten, a process closely associated with the development of various diseases. Telomere length is a risk factor for numerous diseases, including cancer, reproductive disorders, and neurodegenerative conditions. Shortened telomeres correlate with an increased disease risk, highlighting the critical role of telomere dynamics in disease onset and progression. Telomerase, a multi-subunit ribonucleoprotein complex with reverse transcriptase activity [2], functions predominantly to elongate telomeres. Comprising telomerase reverse transcriptase (TERT) and telomerase RNA (TER), telomerase provides the necessary template for telomeric DNA synthesis. Regulation of telomerase activity involves multiple molecular mechanisms; in normal cells, telomerase activity is tightly controlled to stabilize telomere length. In certain cancer cells, however, telomerase activity is aberrantly high, supporting uncontrolled cellular proliferation and immune evasion.

**Figure 1:**
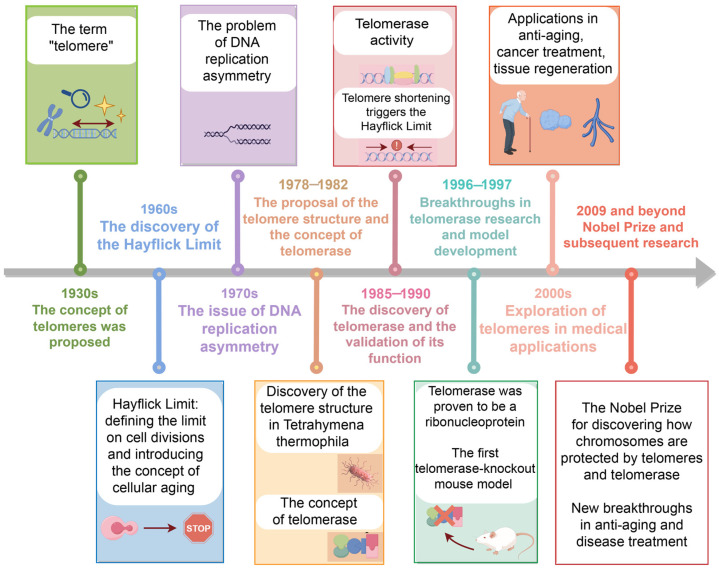
Developmental timeline of telomeres and telomerase. Key advancements in telomere and telomerase research are depicted (created with Figdraw).

Telomerase, the sole enzyme capable of extending telomere length [3], is critical for telomere stability and cellular senescence prevention. In most somatic cells, telomerase expression is minimal; however, in reproductive stem cells and cancer cells, its levels are sufficiently high to sustain telomere length. During tumorigenesis, telomerase activity is markedly upregulated, enabling cancer cells to proliferate indefinitely by continuously repairing and elongating telomeres despite division-induced shortening. Consequently, telomerase activity is a fundamental requirement for cellular immortality, detectable in approximately 90% of malignant tumors. Given telomerase’s pivotal role in cancer, researchers are developing innovative cancer therapies that modulate telomerase activity. These include strategies to inhibit telomerase to curb cancer cell proliferation or, conversely, to activate telomerase in select cells to boost anti-cancer capabilities. The progression of telomere research technologies has traversed several critical milestones (Figure 1).

## 2. Telomere Length Is Closely Related to Life Span and Health Status

**Figure 2:**
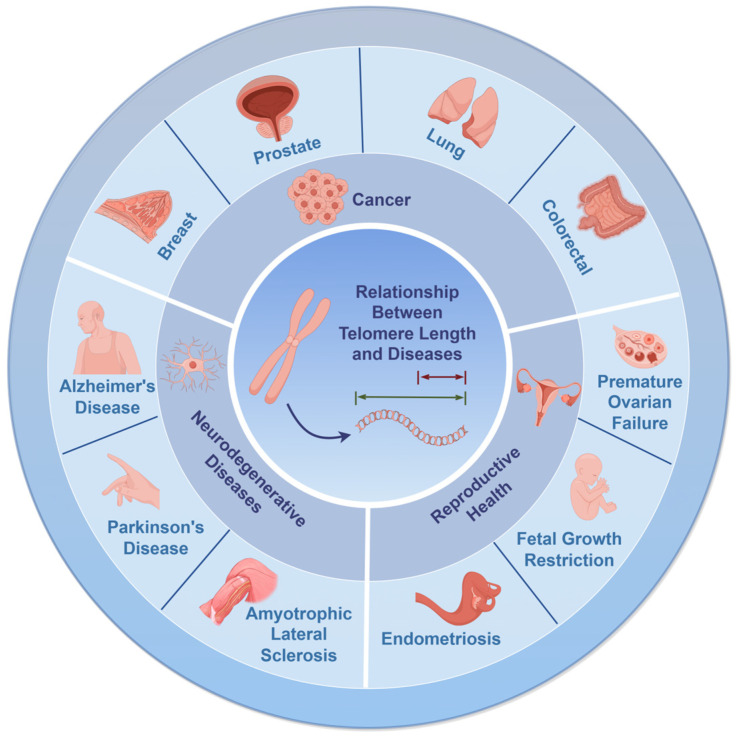
Correlation between telomere length and diseases, covering cancer, neurodegenerative disorders, and reproductive health. White-lined areas highlight specific diseases within each category (created with Figdraw).

Telomere length closely correlates with lifespan and health, particularly in tumorigenesis [4] (Figure 2). Shortened telomeres signal accelerated cellular aging and heightened cancer risk, while maintaining optimal telomere length helps curb abnormal cell proliferation, thereby offering protection against cancer. Adopting a healthy lifestyle and integrating nutritional supplements can mitigate telomere attrition, promoting health and longevity [5].

Telomere length maintenance is fundamentally linked to cancer development, and telomere length variation (TLV) and average telomere length jointly influence lung cancer risk [6]. In blood lymphocytes, TLV significantly correlates with lung cancer susceptibility, moderated by age, suggesting that TLV combined with average telomere length could help identify high-risk groups for lung cancer through computed tomography screening. Numerous studies indicate telomere shortening as a potential lung cancer risk marker, associating telomere attrition in peripheral blood leukocytes with lung cancer and COPD [7,8]. Long telomeres exert a protective effect against squamous cell carcinoma (SCC) [9], particularly in males, suggesting that telomere length impacts lung cancer risk variably across histological subtypes. Associations between telomere length (TL) and cancer risk have been investigated in many epidemiological studies of lung cancer, but the results remain controversial. Longer TL in peripheral blood cells may heighten the risk of lung cancer in men [10], and further studies in Asia found that prolonged TL is associated with an increased risk of lung cancer in women, notably among non-smokers [11,12]. The relationship between mean telomere length in buccal cells and bladder cancer risk showed that patients with bladder cancer exhibited significantly shorter telomeres than controls (*p* = 0.001). The median telomere length ratio was 0.95 (range 0.53–3.2) for cases and 1.1 (0.51–2.4) for controls. Known factors such as oxidative stress, alkylation, and UV radiation contribute to accelerated telomere shortening [13]. Ten LTL-associated SNPs served as instrumental variables, with individual-level data-based weighted genetic risk scores (GRS), and summary statistics-based inverse–variance weighting (IVW) in MR analyses revealed that smokers had shorter telomeres than non-smokers [14]. Pavanello et al. explored how genetic polymorphisms, alongside environmental and occupational exposures, affect LTL and BC risks [15]. Structural equation modeling (SEM) assessed these complex interactions, and smokers with shorter telomeres were at an elevated risk of bladder cancer. Lin et al. identified a relationship between short telomeres and depressive symptoms in conjunction with increased bladder cancer mortality [16], and Yu et al. further observed that short telomeres, combined with *GSTM1* homologous deletion, heightened bladder cancer risk [17]. Utilizing flow cytometry-based fluorescence in situ hybridization, a study observed that more aggressive aneuploid tumors exhibit longer telomere lengths compared to diploid tumors (FISH) [18]. Wang et al. reported that individuals with longer telomeres and greater telomere length variability may have a 14-fold increased risk of developing bladder cancer [19]. Additionally, telomere length variability was elevated in patients with bladder cancer, indicating significant dysregulation in telomere function. These studies consistently show that there is a significant correlation between the telomere length and the risk of cancer, and multiple factors such as smoking, genetic variation, and environmental exposure can affect this relationship.

Patients with short telomeres exhibited significantly reduced treatment-free survival (TFS) and overall survival (OS) compared to those with long telomeres (*p* < 0.001 and *p* = 0.015, respectively) [20], establishing telomere length as an independent prognostic indicator for TFS. Gu et al. reported that individuals with the *RETL1* rs2297441 AA variant had shorter telomeres [21] and an increased prostate cancer risk. Longer telomeres may elevate prostate cancer risk, particularly in genetically predisposed cases and among men with a family history. Conversely, in male heavy smokers without such a family history, shorter telomeres correlated with a reduced prostate cancer risk [22,23,24]. Additionally, telomere length variability was notably higher in African American men with high-grade prostate cancer than in Caucasian men with similar disease severity [25]. Studies have also found that patients with breast cancer often exhibit longer telomeres, with estrogen levels previously shown to influence telomere length. This suggests that menopausal status may impact telomere dynamics, as well as the association with insulin resistance and inflammation [26,27]. Duggan et al. observed no significant association between peripheral leukocyte telomere length and either all-cause or breast cancer-specific mortality when measured at baseline and 30 months post-diagnosis in survivors [28]. However, participants with telomere shortening over this period showed higher breast cancer-specific and all-cause mortality, suggesting that longer telomeres may protect against the break–fusion–bridge cycle, a process that promotes cellular senescence. Notably, cancer cell telomeres measured with quantitative FISH were shorter than those in normal epithelial cells [29].

## 3. Telomere Measurement Methods and Their Advantages and Disadvantages

Identifying the optimal and most sensitive telomere measurement method is essential for advancing strategies to promote healthy cell growth while limiting or preventing cancer cell proliferation [30]. Table 1 summarizes various telomere measurement methods, along with their respective advantages and disadvantages.

### 3.1. Telomere Restriction Fragment Analysis

Telomere length is often measured indirectly via telomere restriction fragment (TRF) analysis, a modified Southern blot technique that assesses the distribution of terminal restriction fragments to gauge telomere length heterogeneity in a cell population.

TRF length analysis leverages the absence of restriction sites within telomeric regions to generate a DNA molecular weight (MW) ladder, with the choice of molecular weight ladder affecting the final telomere length calculation. DNA integrity is verified using agarose gel electrophoresis and visualized with nucleic acid stains. Digested DNA samples are separated by size via agarose gel electrophoresis, where longer telomeric fragments migrate more slowly than shorter fragments [31]. TRF is regarded as an excellent standard for measuring absolute telomere length [32], though it only provides an average telomere length and cannot identify the shortest telomere lengths within a sample.

### 3.2. Polymerase Chain Reaction

Polymerase chain reaction (PCR) is a molecular technique used to amplify specific DNA fragments, effectively enabling in vitro DNA replication [33]. PCR is notable for its ability to significantly increase DNA quantity. However, interindividual variation in centromeric alpha satellite DNA (α DNA) and Arthrobacter luteus DNA (Alu DNA) sequence copy numbers remains unquantified, potentially causing inaccuracies in relative telomere length comparisons across individuals [34]. To address this, telomere measurement using PCR normalizes the number of telomeric repeats to single-copy genes, thereby enhancing accuracy. This PCR-based approach is simple, rapid, and scalable for high-throughput sample processing.

### 3.3. Fluorescence In Situ Hybridization

FISH is a mapping technique that utilizes fluorescein-labeled probes to detect hybridization between probes and chromosomes during metaphase or chromatin in interphase. Quantitative FISH (Q-FISH) employs fluorescence microscopy and digital imaging to measure telomeric repeat length in metaphase chromosomes. The basic Q-FISH technique effectively provides qualitative insights into the presence of specific repeats within the nucleus. The method developed by Poon et al. enables the visualization of all 46 chromosomes in metaphase [35], including chromosomes 1 through 22 and sex chromosomes, allowing chromosome-specific telomere length assessment. However, Q-FISH depends on probe hybridization kinetics, which limits its ability to quantify the shortest telomeres.

Flow FISH combines peptide nucleic acid (PNA) probes with flow cytometry [35] to measure average telomere length while capturing multiparametric data from thousands of cells. This method is primarily applicable to cultured cells or single-entity lymphocytes [36], and is well-suited for estimating average telomere length in interphase cells, especially human lymphocytes. Although an improvement over Q-PCR, Flow FISH requires expensive equipment [37] and is limited by nonspecific probe interactions with cytoplasmic components.

### 3.4. Single Telomere Length Analysis (STELA)

Single telomere length analysis (STELA) combines ligation Q-PCR with Southern blotting, providing precise measurements of individual chromosome telomere lengths. STELA can assess telomere length across chromosomes with known sequences [38], though it is unsuitable for damaged samples.

Universal–single telomere length analysis (U-STELA) is an extension of STELA designed to measure telomere length across all chromosomes, hence termed “universal” STELA [39]. Unlike traditional STELA, U-STELA can assess telomere length genome-wide and is capable of identifying key short telomeres even in low-concentration samples [40]. However, a notable limitation of U-STELA is its difficulty in accurately measuring longer telomeres. High-throughput single telomere length analysis (HT-STELA) leverages PCR amplification to target telomeric regions for high-throughput analysis using instruments such as bioanalyzers [41]. This method streamlines telomere measurement by automating workflows, significantly increasing efficiency and reducing manual steps by removing the need for traditional gel electrophoresis and hybridization. Similar to STELA, HT-STELA can measure telomere length across chromosomes with known sequences and, like U-STELA, requires only minimal DNA input.

### 3.5. Telomere Shortest Length Assay (TeSLA)

TeSLA is a telomere length measurement technique designed to specifically target the shortest telomeres within cells [42], which are essential for regulating cell cycle arrest and mitigating age-related diseases. Unlike previous methods that primarily assess average telomere length, TeSLA represents an advancement from STELA, with the capacity to measure telomeres across all chromosomes simultaneously [43]. In contrast, STELA typically analyzes telomeres on selected chromosomes. A limitation of TeSLA, however, is its inability to pinpoint the shortest telomere on a specific chromosome with precision. Compared to other TL measurement techniques, TeSLA offers greater sensitivity, effectiveness, and specificity in directly detecting TL.

### 3.6. New Detected Methods for TL

The single-cell telomere length measurement method (SCT-pqPCR) is a powerful and simplified approach for assessing telomere length in individual cells [44]. By optimizing multiplex pre-amplification specific to telomeres and reference genes, SCT-pqPCR achieves a consistent telomere-to-reference gene (T/R) ratio via Q-PCR. This method allows for the detection of telomere length in quiescent cells, which quantitative FISH cannot detect. SCT-pqPCR enables telomere measurement in single cells independent of their division capacity and offers a tool to assess telomere length variability across cell populations [45]. Despite these advancements, clinical telomere analysis still faces hurdles due to the lack of simple, rapid, and scalable measurement techniques. The single telomere absolute-length rapid (STAR) assay addresses this with a high-throughput digital real-time PCR method that allows quick measurement of the absolute length and quantity of individual telomere molecules [46]. Optical mapping enables direct visualization and structural analysis of long DNA molecules by aligning DNA within nanoscale channels, labeling it with fluorescent dyes, and observing it with high-resolution microscopy [47].

The telomere length comb assay (TCA) measures telomere length on stretched DNA fibers [48], and it has been applied to assess telomere erosion in primary human fibroblasts and the effects of telomere maintenance pathway activation on telomere elongation. TCA also measures telomere length in healthy individuals and detects critically short telomeres in patients with telomere biology disorders. TCA does not require circulating cells, as it operates on isolated DNA and is compatible with semi-automated or fully automated image analysis using the Genomic Vision molecular combing platform. This approach mitigates sampling bias and supports high-throughput applications and clinical development. TCA is a versatile and straightforward technique that enables detailed telomere length distribution analysis across cell populations, providing enhanced accuracy and biological insight for telomere studies.

**Table 1:** Current major telomere length measurement methods and their advantages and disadvantages.

Methods	Required DNA Volume/ Cells	Results	Experiment Time	Advantages	Limitations	Reference
TRF	>3 µg	average TL	>96 h	- Gold standard - Widely available - Commercialized kit	- Only the average value can be measured, and the shortest length cannot be captured - Requires substantial amount of DNA	[28]
Q-PCR	20 ng	average TL	<2 h	- Simple, fast, and easily scalable, enabling high-throughput sample processing	- Needs to check the single-copy gene of the sample	[31]
Q-FISH	10–15 cells	Shortest TL	>72 h	- Able to measure the shortest TL and TL per chromosome	- Needs high-quality microscope, is time-consuming and expensive	[32]
FLOW-FISH	1 × 10^5^ cells	Average TL	>72 h	- Can be performed in a high-throughput format	- Interpretation challenges - Proper sample preparation is crucial - Time-consuming and expensive	[34]
STELA	10–50 ng	Shortest TL	>72 h	- Able to measure TL on each chromosome of known sequence	- Cannot be used for damaged samples	[35]
U-STELA	10–40 pg	Shortest TL Absolute TL	>72 h	- Able to measure TL on each chromosome - Only a small amount of DNA is required	- Unable to distinguish chromosomes - Measuring telomeres is challenging	[36]
HT-STELA	10–40 pg	Shortest TL Absolute TL (with known sequences)	>72 h	- Able to measure TL on each chromosome of known sequence - Only a small amount of DNA is required	- Can only analyze chromosomes with known subtelomere sequences	[38]
TeSLA	10–40 pg	Shortest TL Absolute TL	>72 h	- All telomeres with chromosome ends less than 1 KB and up to 18 KB were measured - Applicable to numerous animal types	- Low throughput - Labor-intensive	[40]
SCT-pqPCR	10 pg	Average TL	>2 h	- Can detect the telomere length of quiescent cells	- Accuracy in single-cell separation	[41]
STAR Assay	<1 ng	Absolute TL Shortest TL	<3 h	- Rapid measurement of the absolute length and number of individual telomere molecules	- Limited historical data - High cost and complexity	[43]
Optical mapping	20 µg	Shortest TL Absolute TL	>24 h	- Able to measure TL on each chromosome of known sequence	- High cost and complexity	[44]
TCA	1–2 × 10^6^ cells	Shortest TL Absolute TL	>72 h	- Telomeres can be directly visualized and quantified - High precision	- Difficult to perform on large samples - High cost and complexity	[45]

## 4. The Dual Effects of Telomerase Activity Regulation

Telomerase is critical for chromosome stability and cellular longevity, with its activity regulation integral to cellular functionality [49]. Dysregulated telomerase activity is strongly associated with the onset and progression of numerous diseases, particularly cancer, positioning it as a promising therapeutic target. Extensive research may inspire innovative treatment approaches for related diseases. As a broad-spectrum tumor biomarker, telomerase holds potential as a novel target in cancer therapy, with regulatory insights offering substantial value for anti-cancer drug development.

**Figure 3:**
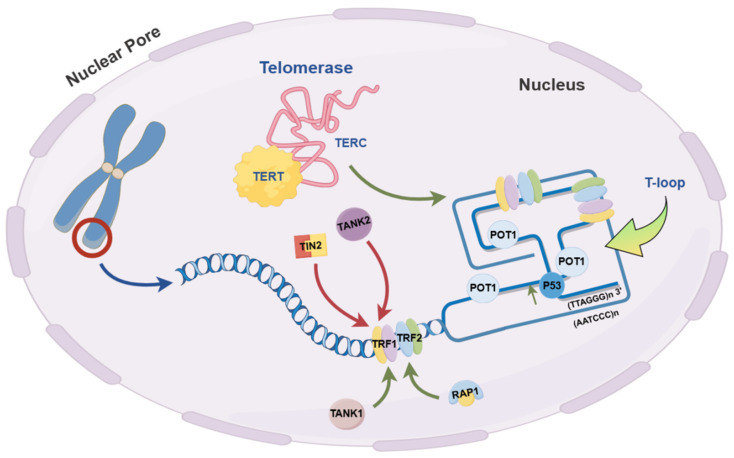
Molecular mechanism of telomere maintenance. Red arrows represent inhibitory effects on telomere length maintenance through binding to target proteins, such as telomerase activity suppression or promotion of telomere degradation. Green arrows denote promoting effects on telomere length maintenance, including the enhancement of telomerase activity or stabilization of telomere structure (created with Figdraw).

Telomerase activity regulation involves a complex, multilevel interaction network that preserves chromosomal stability and cell functionality throughout division. As a ribonucleoprotein complex [3] with reverse transcriptase activity, telomerase extends telomeric repeats (e.g., TTAGGG) at chromosomal ends, counteracting telomere shortening due to cell division—a vital function for sustaining cellular proliferation and chromosome integrity. Certain proteins, such as TPP1, enhance telomerase catalytic activity [2] and interact with other telomere-associated proteins to form complexes that fortify telomere protection. Conversely, inhibitory proteins bind to telomerase to prevent excessive telomere elongation, playing an essential role in telomere length equilibrium [50]. The molecular mechanisms underlying telomere maintenance are illustrated in Figure 3.

Elevated telomerase activity in cancer cells is associated with reduced treatment sensitivity [51], making telomerase a promising cancer therapy target. Guterres et al. discussed recent findings on telomerase, noting that targeting it could have a transformative impact on cancer treatment, with advances in the structural modeling of human telomerase enhancing prospects for clinically effective drugs [52]. Eitsuka et al. reviewed the regulatory effects of dietary compounds on cancer cell telomerase activity [53], reporting telomerase expression in approximately 90% of human cancer cell lines and tumors, and its absence in most somatic cells, underscoring its potential as a selective cancer therapy target. Mizukoshi et al. focused on hTERT-based immunotherapy, finding that telomerase activity enables cancer cells to proliferate uncontrollably, infiltrate tissues, and metastasize to distant organs [54]. Greider et al. examined telomerase activity and cell proliferation in cancer, suggesting that telomerase could serve as a diagnostic marker for staging in various cancers [55]. Regulating telomerase activity is essential for normal cellular functions. In stem and germ cells, controlled telomerase activity supports ongoing division and chromosomal stability, while most somatic cells suppress telomerase to prevent hyperproliferation and tumorigenesis. Under cellular stress or damage, telomerase regulation may shift to protect cells from injury.

The regulatory mechanisms by which non-coding RNAs (ncRNAs) modulate telomeres are intricate and multifaceted [56]. Among these, long non-coding RNAs (lncRNAs), such as TERRA, are directly transcribed from telomeric DNA. They interact with telomere-binding proteins, influencing telomere structure and function to regulate telomere length and stability. Additionally, microRNAs (miRNAs) indirectly control the expression of critical telomere-associated proteins, such as telomerase, by targeting mRNAs essential for telomere maintenance [57]. Through inhibiting or promoting the translation of these mRNAs, miRNAs contribute to the dynamic balance of telomere length. ncRNAs also participate in epigenetic processes like DNA methylation and histone modification, further impacting the chromatin state of telomeric regions. Collectively, ncRNAs precisely modulate telomeres through multiple pathways, supporting genome stability and the normal physiological functions of cells.

## 5. Telomere Length Maintenance and Reproductive System Diseases

Telomeres regulate biological aging across plants, yeast, mammals, and other organisms [58]. The telomere theory of reproductive aging posits that telomere shortening in female germ cell lines is a primary driver of reproductive aging in females [59]. Male germ cells, rich in spermatogonia, exhibit stable or even lengthening telomeres with age, whereas female ovaries lack germline stem cells, causing oocytes to experience pronounced aging. This process ultimately leads to genomic instability, spindle abnormalities, and embryonic defects that result in cell cycle arrest, fragmentation, and apoptosis [60]. Increasing evidence links telomere-related changes to endometriosis and gynecologic cancers, both conditions affecting the female reproductive system [61]. During germ cell meiosis, telomeres not only protect chromosomes from damage but also regulate critical movements such as chromosome alignment, pairing, and crossover. These steps are essential for producing sperm and eggs with a normal chromosomal profile. Damage to telomere structure can induce germ cell aging, function loss, and infertility [62]. Telomerase is vital for telomere length maintenance, with significant expression during embryogenesis and sustained high activity in germ cells, stem cells, and cancer cells. However, telomerase activity in somatic cells diminishes after the neonatal period. Dysregulated telomerase activity can impair germ cell quality and embryonic development, potentially causing infertility or developmental anomalies [63]. For instance, in the female reproductive system, telomere length and telomerase activity are indicators of oocyte quality and aging. In endometrial cells, telomerase activity and telomere length dynamics are essential for pregnancy, with abnormal elevations potentially linked to recurrent miscarriages (RM). In endometriosis, longer telomeres and higher telomerase activity in ectopic endometrial cells may contribute to the disease pathogenesis.

To assess the association between cellular senescence markers and reproductive failure [64], telomerase expression and telomere length were examined in endometrial biopsies from women with and without fertility issues. Quantitative polymerase chain reaction determined the average endometrial telomere length, revealing minimal telomerase immunoreactivity in the control group’s endometria during the implantation window. Conversely, women with recurrent fertility failure exhibited significantly elevated and variable telomerase immunostaining across different endometrial cell compartments (*p* < 0.05), though no significant difference in average telomere length was observed between the groups. In a cross-sectional study, Butts et al. identified a link between absent telomerase activity in granulosa cells and occult ovarian insufficiency [65]. Women with this condition demonstrated shorter telomeres compared to controls, suggesting that disrupted telomere homeostasis is implicated in occult ovarian insufficiency among young women. Hapangama et al. (2010) analyzed ectopic active peritoneal endometriosis lesions from seven symptomatic women, finding that while all lesions expressed proliferation markers, they exhibited weak or absent γ-H2AX staining, implicating endometriosis in these cellular changes [66]. Hanna et al. utilized telomere-specific quantitative PCR to measure telomere length in two cohorts of women exhibiting reproductive aging markers, concluding that women with RM may present with shorter telomeres, potentially due to accelerated aging or heightened cellular stress [67]. The longer telomere length observed in the premature ovarian failure (POF) group may result from abnormal hormone exposure, a reduced cell division rate, or autoimmune factors. Despite the limited sample size, these findings indicate that distinct manifestations of reproductive aging may be influenced by varied physiological factors. Most high-grade serous carcinomas (HGSCs) display shorter telomeres compared to normal fallopian tube epithelial cells [68]. Treff et al. identified an association between telomere DNA deficiency and somatic genomic instability, suggesting that telomere deficiencies may contribute to the aneuploidy frequently seen in female germ cells and human embryos [69]. Compared to other histological types, clear cell carcinoma exhibits longer average telomere length, which correlates with increased mortality, implying a significant role for telomere length aberrations in the tumor’s onset and progression [70]. Ectopic endometriosis lesions show markedly increased telomerase activity, with extended mean TLS and reduced steroid receptor gene expression [71]. In studying individual human oocytes, sperm, and male and female pronuclei to assess parental contributions to zygote telomere length, direct telomere length comparisons reveal that male telomeres are generally shorter than female telomeres at fertilization. This suggests that telomere length may be influenced by recombination events in oocytes before telomerase activity rises at the blastocyst stage [72]. Relative telomere length, measured against a single-copy gene marker (36B4), showed that mature oocytes have longer telomeres than immature ones, and high-quality embryos exhibit longer telomeres compared to lower-quality embryos [73]. Additionally, telomeres in patients with endometriosis are longer than those in patients without the condition [74].

## 6. Telomere Length Maintenance and Neurodegenerative Diseases

A significant association exists between telomere length and neurodegenerative diseases. Telomeres, specialized structures at eukaryotic chromosome ends composed of DNA sequences and proteins, are essential for maintaining chromosome integrity and stability. With each cell division, telomeres progressively shorten; once they reach a critical length, cells cease division and undergo senescence or apoptosis [75]. Recent studies highlight a strong link between telomere length and neurodegenerative conditions like Alzheimer’s disease (AD) [76]. For instance, individuals with shorter telomeres have an elevated risk of AD and other cognitive impairments, suggesting telomere length as a potential biomarker for assessing neurodegenerative disease risk. Further research has associated longer telomeres with improved brain health, including greater total gray matter volume, larger hippocampal size, and thicker cerebral cortex—all regions typically atrophied in patients with AD. This correlation implies that longer telomeres may offer a protective effect against neurodegenerative decline. Moreover, telomere length is implicated in the biological mechanisms underlying neurodegeneration. Telomere shortening, for example, is linked to mitochondrial dysfunction and increased oxidative stress, both critical factors in the pathogenesis of neurodegenerative diseases [77]. Thus, interventions aimed at maintaining telomere length may introduce novel strategies for neurodegenerative disease treatment.

Telomeres, often referred to as the “protective cap” of chromosomes, undergo shortening with each cell division due to end-replication issues—a process known as telomere attrition, which is linked to various age-related diseases, including AD. Kuan et al. explored key factors that regulate telomere length and preserve its integrity [78], with a particular focus on oxidative stress, commonly associated with aging and molecular damage. Despite some conflicting findings, telomere attrition is believed to play a vital role in AD progression due to its strong correlation with oxidative stress. Crocco et al. suggested that, during aging [79], telomere shortening, indicative of premature cellular aging, may contribute to age-related diseases such as AD. This has led some studies to hypothesize that telomere shortening could be a characteristic feature of AD. However, the relationship between leukocyte telomere length (LTL) and AD risk remains inconclusive. Current findings nonetheless support LTL shortening as a potential indicator of AD pathogenesis. Hsiao et al. studied telomere length in peripheral blood mononuclear cells (PBMCs) from HIV-1 infected rapid progressors (RPs) on combined antiretroviral therapy (cART) versus cART-naïve RPs [80]. Results showed significant telomere reduction in cART-treated RPs compared to cART-naïve cases. Additionally, HIV Tat-treated human microglia exhibited decreased telomerase activity, telomere length, and mitochondrial function alongside elevated oxidative stress. These findings suggest that neurocognitive impairment in HIV may partly arise from accelerated neuropathogenesis in microglia, attributed to heightened oxidative stress and mitochondrial dysfunction.

## 7. Challenges in Telomere Length Maintenance Research and Future Research Directions

Maintaining telomere length, particularly countering telomere shortening, presents numerous challenges but also offers promising research avenues. Measures that can be taken to reduce telomere shortening have been recorded. Telomere shortening is a complex, multifaceted process involving DNA replication, telomerase activity, epigenetic modifications, and noncoding RNA regulation, among others [81]. These elements create an intricate regulatory network, complicating efforts to fully understand telomere shortening mechanisms. The rate and pattern of telomere shortening vary significantly among individuals, tissues, and cell types, influenced by factors such as genetic background, lifestyle, and environmental exposures, adding further complexity to this field of study. Mitigating telomere shortening requires a comprehensive health approach. A balanced diet forms the foundation, complemented by regular, moderate exercise, which supports telomere maintenance. Additionally, avoiding smoking and excessive alcohol intake is crucial, as these behaviors can accelerate telomere attrition. Routine health check-ups and the timely management of chronic conditions further support overall health and indirectly safeguard telomeres. Together, these strategies may slow telomere shortening and delay aging. However, the dynamic nature of telomere length poses challenges in translating research findings into clinical diagnostics and treatments. Despite advancements in biotechnology, the accurate detection and quantitative analysis of telomere shortening face technical constraints. Highly sensitive and precise methods are necessary for telomere measurement, yet practical factors like sample quality and experimental conditions often impact accuracy. The close link between telomere shortening, aging, and disease also raises ethical considerations. Researchers must balance scientific progress with human welfare, ensuring fairness and accessibility in applying findings. These ethical dimensions warrant careful attention as telomere research progresses.

Future research should intensively explore the molecular mechanisms and regulatory networks driving telomere shortening, focusing on emerging regulatory elements like ncRNA and epigenetic modifications. Utilizing advanced technologies such as high-throughput sequencing, single-cell sequencing, and other state-of-the-art methodologies [82], researchers can clarify the specific functions and regulatory pathways of these factors within the telomere shortening process. Given the strong association between telomere shortening and the onset and progression of various diseases, future studies should rigorously investigate the links with cardiovascular diseases, neurodegenerative disorders, tumors, and other conditions. Such research will be instrumental in revealing the roles and mechanisms of telomere shortening in these disease pathways.

## Data Availability

Data supporting the findings of this study are available from the corresponding author upon reasonable request.
